# Acute Onset of a Life-Threatening Skin Toxicity Due to Osimertinib: Severe Psoriasis Versus Toxic Epidermal Necrolysis

**DOI:** 10.7759/cureus.24513

**Published:** 2022-04-26

**Authors:** Rebekah Rittberg, Cheryl Ho, Ying Wang

**Affiliations:** 1 Medical Oncology, BC Cancer, Vancouver, CAN; 2 Medicine, The University of British Columbia, Vancouver, CAN

**Keywords:** treatment related toxicity, non-small cell lung cancer (nsclc), rare side effect, plaque psoriasis, stevens-johnson syndrome (sjs), toxic epidermal necrolysis (ten), egfr mutations, skin and mucosal toxicity

## Abstract

Osimertinib is a third-generation irreversible epidermal growth factor receptor (EGFR) tyrosine kinase inhibitor currently used as first-line systemic therapy for advanced EGFR mutant non-small cell lung cancer. Osimertinib is generally very well tolerated with only a 1% risk of grade 3-4 skin toxicity. Here we present a case of a 68-year-old Asian male with advanced EGFR exon 19 deletion non-small cell lung cancer. After initiation of osimertinib 80 mg daily, he had a rapid worsening of his pre-existing scaly psoriatic plaques with desquamation. Treatment was withheld while psoriasis therapy was administered. He was rechallenged on osimertinib 40 mg daily and within three days developed fever, tachycardia and widespread skin desquamation. There was an initial concern of toxic epidermal necrolysis; however, this was ultimately determined to be a severe flare of psoriasis. This case serves as a reminder that severe and potentially life-threatening complications can occur, and it is imperative to maintain a high level of vigilance for unusual toxicities of EGFR tyrosine kinase inhibitors, including Stevens-Johnson Syndrome (SJS) and toxic epidermal necrolysis or psoriasis.

## Introduction

Osimertinib is a well-tolerated third-generation irreversible epidermal growth factor receptor (EGFR) tyrosine kinase inhibitor (TKI) currently used as first-line systemic therapy for advanced non-small cell lung cancer (NSCLC) harbouring EGFR sensitizing (exon 19 deletions and L858R) mutations or EGFR resistant (T790M) mutations. Osimertinib is generally very well tolerated, with only 34% of patients experiencing a grade ≥3 toxicity. Skin toxicity is a common toxicity; however, generally mild, with dry skin, acne or rash being most commonly reported [1]. Here we present a case of a very severe, potentially life threatening skin complication to osimertinib.

## Case presentation

A 68-year-old Asian male, lifelong non-smoker, was diagnosed with recurrent NSCLC. He had a 30-year history of psoriasis previously successfully treated with UVB (type B ultraviolet) phototherapy. He originally presented with left upper lobe Stage IIB (pT3 (7.8cm) N0 (0/12)) adenocarcinoma, underwent lobectomy and received four cycles of adjuvant cisplatin and vinorelbine at the age of 66. Surveillance imaging identified multifocal ground glass nodules in lungs bilaterally, initially one year after adjuvant chemotherapy, which showed interval growth over multiple scans. Molecular characterization identified an epidermal growth factor receptor (EGFR) exon 19 deletion. 

First-line osimertinib 80 mg daily was started one-and-half years after completion of adjuvant chemotherapy. Within two weeks, the patient had significant worsening of his pre-existing scaly psoriatic plaques with desquamation over his face, trunk, upper and lower extremities, including inflammation of his fingertips with intermittent abdominal pain. Osimertinib was held, and Dermatology prescribed hydrocortisone cream and UVB phototherapy, given the patients previous success receiving these treatments for his psoriasis. Despite this, his rash worsened, and four weeks after osimertinib was held, he developed a temperature of 39.5°C with tachycardia and worsening abdominal pain. He was admitted to hospital, infection was ruled out, and skin toxicity was deemed psoriasis worsened by osimertinib potentially with associated dermatitis. After hospital discharge, he commenced a 10-week course of UVB phototherapy with 90% clearance of skin lesions. 

**Figure 1:**
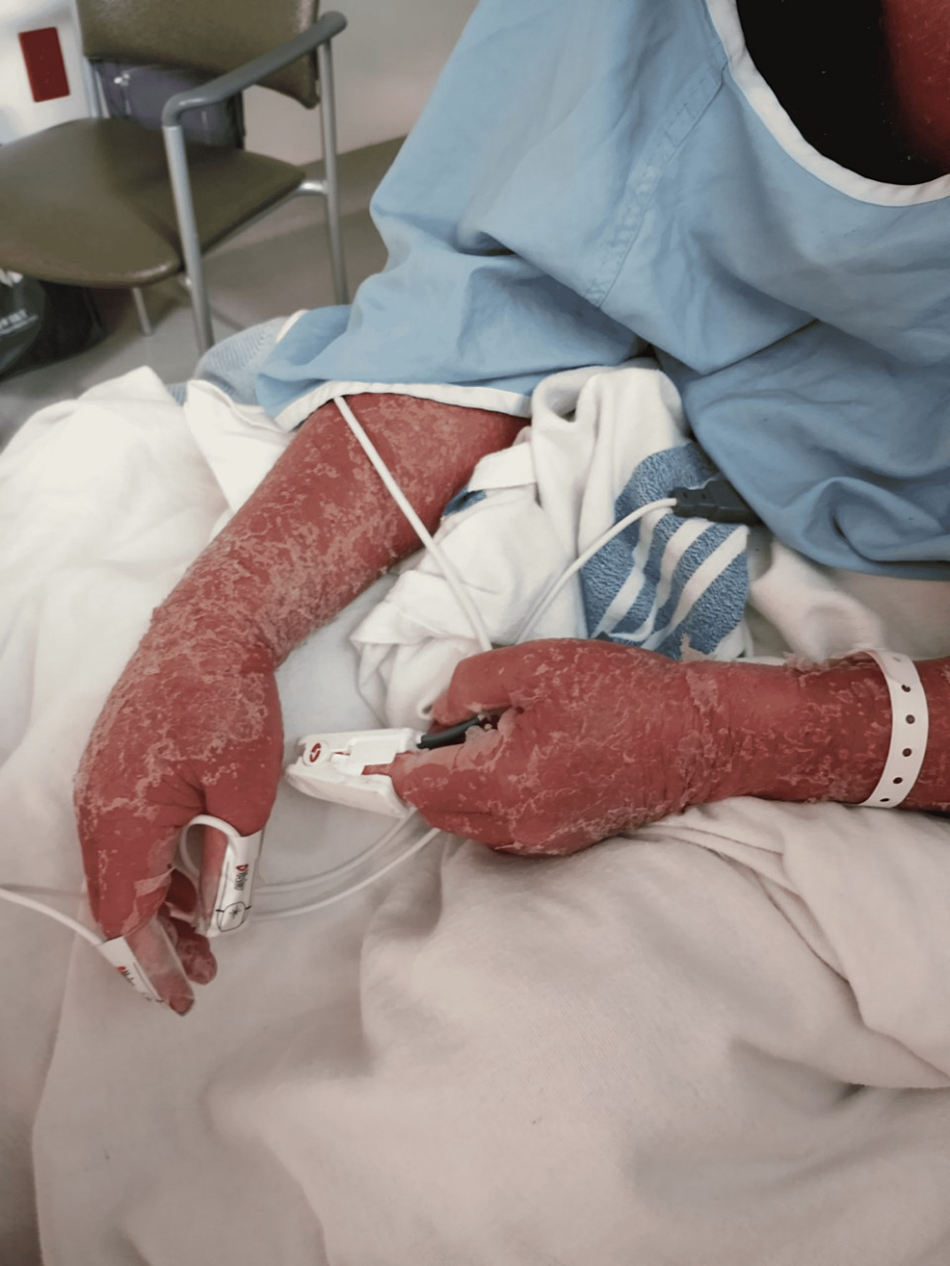
Desquamation of his skin on arms after rechallenging with Osimertinib at 40 mg daily.

**Figure 2:**
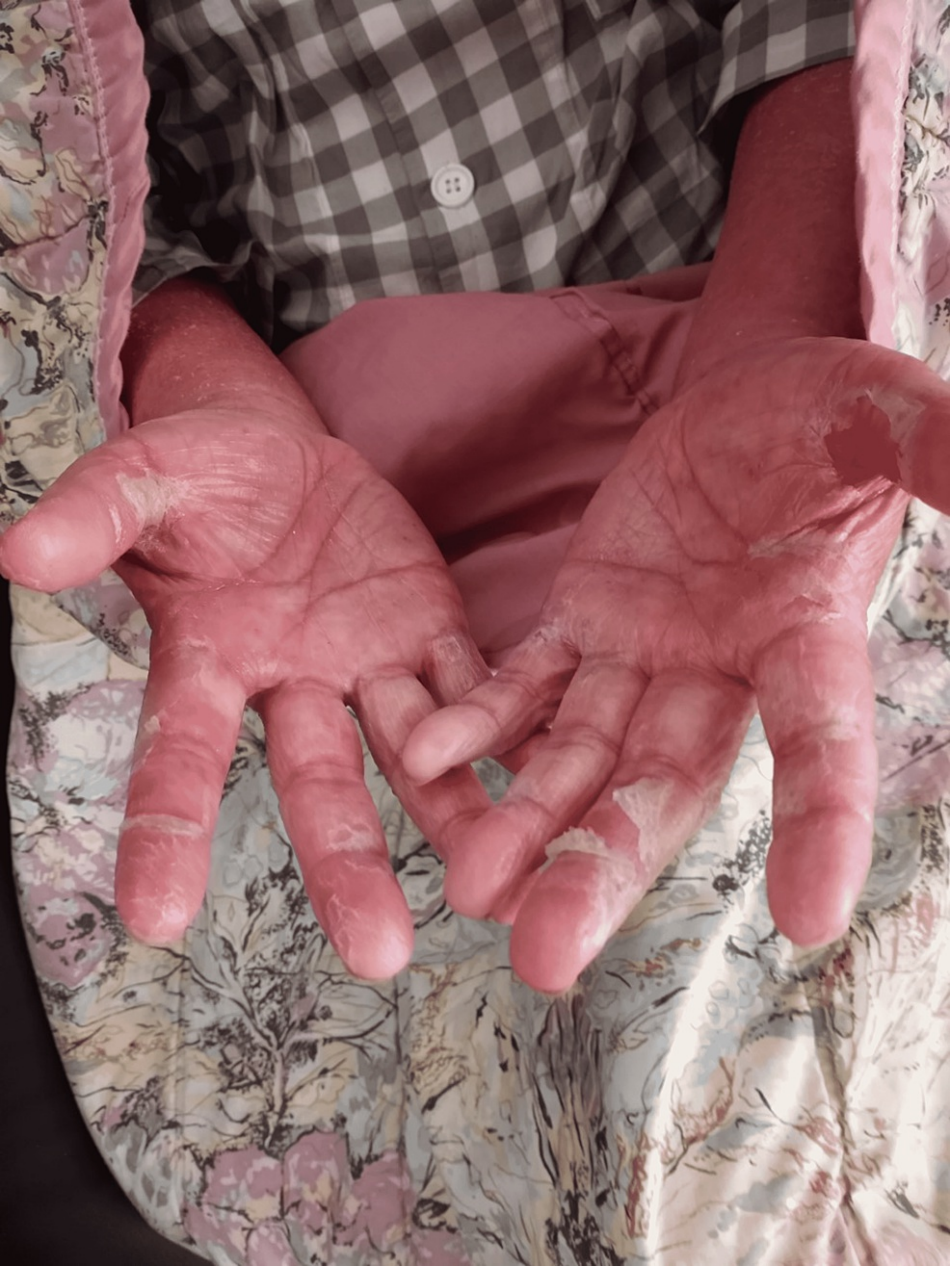
Desquamation over hands after rechallenging with Osimertinib at 40 mg daily.

Osimertinib was restarted at 40 mg daily, after completion of phototherapy, five months since his last dose. Within three days, he developed fever, tachycardia, widespread skin desquamation and swelling, Figures 1, 2.

He did not have mucosal surface involvement. He was found to have a WBC of 18.5 and CRP of 154. Infection was again ruled out, and skin toxicity was deemed to be a severe toxic desquamating psoriatic skin reaction to osimertinib; however, the possibility of toxic epidermal necrolysis (TEN) was raised. After recovery, further challenge with osimertinib was deemed high risk, and he was started on carboplatin and pemetrexed, which has been tolerated without further skin toxicity. 

## Discussion

Osimertinib is generally a well-tolerated third generation irreversible EGFR TKI found in the FLAURA trial to improve survival in the first-line setting compared to gefitinib or erlotinib [1]. Skin toxicity is common with TKIs, with 57% of patients receiving osimertinib experiencing grade 1-2 rash, with only 1% experiencing grade 3-4 [1]. The most common skin manifestations include: acneiform rash, erythema, pruritis, skin cracking, and paronychia [2]. Dermatologic toxicity are frequently observed since EGFR TKI will also target EGFR located in skin epithelium; however, are more frequent in first or second generation EGFR inhibitors [2]. 

Toxic epidermal necrolysis (TEN) and Stevens-Johnson Syndrome (SJS) are very rare life-threatening toxic skin reactions to drugs. To date there are only three cases of TEN and one case of SJS reported from osimertinib [3,4]. In our patient, biopsy was not completed to definitively rule out TEN/SJS, as he was demonstrating clinical improvement. Although a biopsy would have confirmed the underlying skin toxicity, it was felt not necessary by Dermatology. Ultimately, our case was felt to represent an extreme exacerbation of psoriasis due to osimertinib. Exacerbation of psoriasis is another rare toxicity, which accounts for 8% of skin toxicity in first or second generation EGFR TKIs; however, rarely reported from osimertinib [5]. 

## Conclusions

Skin toxicity is a very common side effect of EGFR tyrosine kinase inhibitors, more common in first or second generation than third generation therapies. The most common presentation of skin toxicity in third generation EGFR TKI are grade 1-2. Here we present a case of a severe skin toxicity to osimertinib, which responded to UVB phototherapy. EGFR TKI rechallenge may be reasonable when no other systemic therapy treatment options are available if approached cautiously, as recurrence may occur even after significant dose reduction and it may be life threatening. This case serves as a reminder to maintain a high level of vigilance for unusual toxicities of EGFR TKIs, including SJS/TEN or psoriasis, and highlights the importance of a cutaneous biopsy whenever the diagnosis is not certain. 

## References

[1] Soria JC, Ohe Y, Vansteenkiste J (2018). Osimertinib in untreated EGFR-mutated advanced non-small-cell lung cancer. N Engl J Med.

[2] Liu HB, Wu Y, Lv TF, Yao YW, Xiao YY, Yuan DM, Song Y (2013). Skin rash could predict the response to EGFR tyrosine kinase inhibitor and the prognosis for patients with non-small cell lung cancer: a systematic review and meta-analysis. PLoS One.

[3] Sato I, Mizuno H, Kataoka N (2020). Osimertinib-associated toxic epidermal necrolysis in a lung cancer patient harboring an egfr mutation—a case report and a review of the literature. Medicina (Kaunas).

[4] Li W, He X, Liu H, Zhu J, Zhang HM (2021). Successful treatment after toxic epidermal necrolysis induced by AZD-9291 in a patient with non-small cell lung cancer: a case report. World J Clin Cases.

[5] Annunziata MC, Ferrillo M, Cinelli E, Panariello L, Rocco D, Fabbrocini G (2019). Retrospective analysis of skin toxicity in patients under anti-EGFR tyrosine kinase inhibitors: our experience in lung cancer. Open Access Maced J Med Sci.

